# Comparing Mid-Upper Arm Circumference With Body Mass Index for Assessing Nutritional Status in Indian Adults: Evidence From the National Family Health Survey 2015-16 (NFHS-4)

**DOI:** 10.7759/cureus.59629

**Published:** 2024-05-04

**Authors:** Shailender Negi, Nagapurkar Srinath, Mykala Akshay

**Affiliations:** 1 Public Health, Indian Institute of Public Health, Hyderabad, Hyderabad, IND

**Keywords:** nfhs-4, mid-upper arm circumference, body mass index, anthropometry, malnutrition

## Abstract

Background

The surge in the twin burden of malnutrition - undernutrition and overweight/obesity - poses a severe threat worldwide including India. The adult group, primarily considered as an economic pillar of the society, suffered significant health problems, yet their nutritional issues are often neglected. Screening of nutritional status through anthropometric measurements is widely accepted. Body mass index (BMI) is commonly used but has certain limitations. Mid-upper arm circumference (MUAC), another simpler tool, is universally accepted in children, but its use in adults is debatable. The current research aims to determine the MUAC cutoffs and their predictive accuracies corresponding to BMI cutoffs for adult men and nonpregnant women.

Subject and methods

A cross-sectional analysis was conducted of the anthropometric data of Indian adult men and nonpregnant women collected in 2015-16 via the National Family Health Survey (NFHS-4). The receiver operating characteristic (ROC) curve analysis was performed to derive the MUAC cutoffs against BMI cutoffs.

Results

A significant moderate correlation for both men (r=0.56) and women (r=0.68) was observed. In relation to ROC analysis, the MUAC cutoffs against the BMI cutoffs of 18.5, 23, 25, and 30 kg/m^2^ were approximated to be 25, 26, 28, and 30 cm for men and 23, 25, 27, and 28 cm for women, respectively. These MUAC cutoffs showed good predictive accuracy with a high range of sensitivity and specificity for both men and women.

Conclusions

The non-invasive MUAC method correlates very well with BMI and offers several advantages, including accuracy, ease of measurement, and minimal logistical support and training, and can assess the nutritional status even in geographically remote areas. Therefore, it can be an important tool in public health, especially in resource-limited settings, for identifying populations at risk of malnutrition.

## Introduction

Nutrition plays a critical role in ensuring the comprehensive growth of the population [1]. The concepts of life expectancy and healthy aging are chiefly determined by nutrition [2]. A universal nutritional transformation, as claimed by the World Bank, is undergoing, causing rapid changes in the food system, environments, and standard of living. As a result, many economically developing countries are facing an increase in the burden of overweight and obesity, which was previously thought to be a disease that only affected wealthy nations. Additionally, the coexistence of undernutrition with overweight/obesity poses a serious threat to low- and middle-income countries (LMICs) [3]. With the rise in the prevalence of the twin burden of malnutrition, especially in LMICs, the adult populations of developing nations, including India, have suffered major health problems [3].

The adult group is primarily considered an economic pillar of society, yet their nutritional problems are often ignored [4,5]. Globally, there are 390 million underweight adults, while 2.5 billion are overweight including 890 million obese individuals [6]. According to the Global Hunger Index, India is ranked 111th out of the 125 nations, indicating a significant level of hunger leading to undernourishment [7]. The proportions of underweight and overweight/obese individuals were 22.4% and 18.4%, respectively [8]. Although malnutrition is one of the major yet least-concerned development issues in India, only a few studies have evaluated adults’ nutritional status, as the majority of these focused on children younger than five years [9].

According to the WHO, the use of anthropometric measurements (physical attributes and overall makeup of the body) for assessing the nutritional and health status of adults is widely established. Height, weight, body mass index (BMI), waist circumference, calf circumference, mid-upper arm circumference (MUAC), waist-to-hip ratio, triceps thickness, and subscapular skinfolds are several anthropometric tools [10]. Although these methods are thought to be less accurate for determining nutritional outcomes, their ease of use and lower implementation cost make them the ideal choice for community-based assessments [11,12].

The advantage of being noninvasive, economical, and training-friendly makes BMI, an indicator of generalized adiposity and can be computed by dividing the weight (in kilograms) by the square of height (in meters), as the most frequently used measurement tool [13]. In addition to dietary status, BMI also indicates the socioeconomic status of the adult population in developing countries [14]. Another useful indicator that can efficiently determine nutritional status is MUAC, which measures the upper arm circumference at the midpoint between the tip of the shoulder (acromion processes) and the tip of the elbow (olecranon process) [5]. The measurements can be taken with a flexible, non-stretchable tape (MUAC tape) and can even detect minute variations in subcutaneous fat or muscle mass. Additionally, there is no requirement for expensive equipment or a skilled workforce [15].

However, BMI has certain limitations, especially in injury, impairment, diseased, or differently abled persons [16]. Additionally, the additional weight of the fetus, other products of conception, and added maternal tissue during pregnancy make BMI an inappropriate measurement tool in pregnant women. Furthermore, precise weight and height measurements call for substantial logistical mobilization even in resource-constrained health settings as well as population-based surveys [5].

In children, the use of the MUAC tool in determining malnutrition is accepted worldwide, including LMICs [17]. Although previous literature revealed the efficiency of MUAC in determining adult undernutrition - equal or even superior to BMI - evidence from large and representative samples is still lacking [5,18]. The current research aims to determine the MUAC cutoffs and their predictive accuracies corresponding to standard BMI cutoffs for adult men and nonpregnant women in India.

## Materials and methods

Data source

The current research used data from the National Family Health Survey 2015-16 (NFHS-4), which is equivalent to demographic and health surveys conducted in numerous nations worldwide. For the first time, the NFHS included all six union territories in addition to the 29 states and provided estimates of most indicators of population, health, and nutrition at the district level for each of the 640 districts in the nation based on the results of the 2011 census. The NFHS-4 employed a stratified multistage random sampling procedure. Information regarding the sampling technique and survey questionnaires is available from the NFHS-4 report [6].

Study design

Cross-sectional analysis was conducted of anthropometric data of a nationally representative household sample of Indian adult men and nonpregnant women collected in 2015-16 via the NFHS-4.

Study population and sample size

The NFHS-4 gathered data on 601,509 households, 112,122 men (aged 15-54), and 699,686 women (aged 15-49 years). All adults (men and nonpregnant women possessing BMI data) whose current age was between 20 and 54 years were included in the study. A total of 92,473 men and 435,454 women constituted the study sample after accounting for exclusion criteria.

Exclusion criteria

Pregnant women and those who had given birth two months before the survey were excluded from the study.

Variables used in the study

Anthropometric variables included age, height, weight, BMI, and MUAC. Sociodemographic variables included place of residence, religion, caste/tribe, education level, marital status, source of drinking water, type of toilet, and wealth Index. The choice of variables was guided by published literature.

Statistical analysis

All data analyses were performed using Microsoft Excel and R (R Foundation for Statistical Computing, Vienna, Austria). The distribution of demographic characteristics and anthropometric measurements were determined using descriptive analysis. The strength of the association between BMI and MUAC was determined using correlation analysis. The receiver operating characteristic (ROC) curve analysis was performed to derive the MUAC cutoffs against BMI cutoffs (considering it standard). The optimal cutoff points for MUAC were estimated using Youden’s index [19]. Moreover, the predictive accuracies of the derived MUAC cutoffs were assessed by determining sensitivity, specificity, likelihood ratio positive (LR+) and likelihood ratio negative (LR-), and total misclassification percentage against the corresponding BMI cutoff.

Ethical approval

The present study used data from the NFHS-4, which is a publicly available dataset with no identifiable information on the survey participants. All the ethical concerns, including informed consent, were strictly followed in the NFHS-4. The protocol was reviewed and approved by the Institutional Ethics Committee (IEC) of the Indian Institute of Public Health, Hyderabad, Hyderabad, Telangana, India (IIPHH/TRCIEC/230/2020).

## Results

Although NFHS-4 gathered data on 112,122 men and 699,686 women, complete anthropometric information was available for 92,473 men and 435,454 nonpregnant women. The average age of adult men and nonpregnant women was 34.7 years (standard deviation [SD] of 9.6 and 7.9, respectively). Approximately two-thirds of the participants were urban dwellers, and the vast majority were Hindus belonging to other backward classes. With respect to education, men performed better than women; 70% (65,536/92,473) of the men completed a secondary and higher level of education compared to less than 50% (2,06,090/4,35,454) in the case of women. Predominantly, 75.7% (70,019/92,473) of men and 94.2% (4,10,542/4,35,454) of women were married. Although four out of five participants had access to safe drinking water, one-third of the participants still lacked access to toilet facilities or preferred open defecation. The mean BMI for men and women was 22.2 kg/m^2^ (SD 3.8) and 22.4 kg/m^2^ (SD 4.4), respectively. The mean MUAC for men was significantly higher than that for women (men: 26.9 ±3.1; women: 25.4 ±3.2) (p<0.001). The sociodemographic and anthropometric details of the respondents are given in Table 1.

**Table 1:** Sociodemographic characteristics and anthropometric measurements of the respondents (adult men and nonpregnant women). The data are represented as N, n, %, and mean ± SD. Source of drinking water: “Others” includes rainwater, dug well, spring, pond, river, canal, irrigation channel, tanker truck, etc. Type of toilet: “Others” includes composting toilet, dry toilet, etc. Wealth Index: “Poor” includes poorer and poor, whereas “Rich” includes rich and richer. N, total participants; n, subpopulation; %, percentage; SD, standard deviation

Characteristics	Categories	Men (N=92,473), f (%)	Women (N=4,35,454), f (%)
Place of residence	Rural	28,995 (31.4)	123,509 (28.3)
	Urban	63,478 (68.6)	311,945 (71.6)
Religion	Hindu	68,695 (74.2)	330,919 (75.9)
	Muslim	12,554 (13.5)	53,533 (12.2)
	Others	11,224 (12.1)	51,002 (11.7)
Caste/Tribe	Scheduled caste	16,487 (18.7)	77,703 (18.7)
	Scheduled tribe	16,822 (19.1)	76,421 (18.3)
	Other backward class	35,311 (40.2)	172,642 (41.5)
	None of the above	19,215 (21.8)	88,869 (21.3)
Education level	No education	13,637 (14.7)	163,281 (37.5)
	Primary	13,300 (14.3)	66,083 (15.1)
	Secondary	49,204 (53.2)	172,655 (39.6)
	Higher	16,332 (17.6)	33,435 (7.6)
Marital status	Single	20,843 (22.5)	599 (0.1)
	Married	70,019 (75.7)	410,542 (94.2)
	Divorced/widowed/separated	1,606 (1.7)	24,313 (5.5)
Source of drinking water	Piped water (own/community)	43,190 (46.7)	185,020 (42.4)
	Handpump (own/community)	33,840 (36.5)	167,928 (38.5)
	Others	15,443 (16.7)	82,506 (18.9)
Type of toilet	No toilet/open defecation	32,185 (34.8)	161,315 (37.1)
	Own flush toilet	49,918 (53.9)	215,167 (49.4)
	Own pit toilet	8,596 (9.3)	41,511 (9.5)
	Others	1,774 (1.9)	17,461 (4.0)
Wealth index	Poor	34,048 (36.8)	178,699 (41.1)
	Middle	20,032 (21.6)	91,068 (20.9)
	Rich	38,393 (41.5)	165,687 (38.0)
Age (years)	Mean (±SD)	34.7 (9.6)	34.7 (7.9)
Weight (kg)	Mean (±SD)	59.7 (11.3)	51.9 (11.0)
Height (cm)	Mean (±SD)	163.6 (7.1)	152.1 (6.1)
Body mass index (kg/m^2^)	Mean (±SD)	22.2 (3.8)	22.4 (4.4)
Mid-upper arm circumference (cm)	Mean (±SD)	26.9 (3.1)	25.4 (3.2)

Relationship between BMI and MUAC

Pearson’s correlations were calculated to assess the strength and direction of the linear relationship between MUAC and BMI. The present study found a statistically significant positive relationship (p<0.0001), as confirmed by a moderate correlation coefficient for both men (r=0.56, p<0.001) and women (r=0.68, p<0.001) (Table 2).

**Table 2:** Correlation analysis between MUAC and BMI among study participants at 1% level of significance (p<0.01). BMI, body mass index; MUAC, mid-upper arm circumference

	Pearson correlation (two-tailed)	P-value
Men	0.56	0.000
Women	0.68	0.000

**Figure 1:**
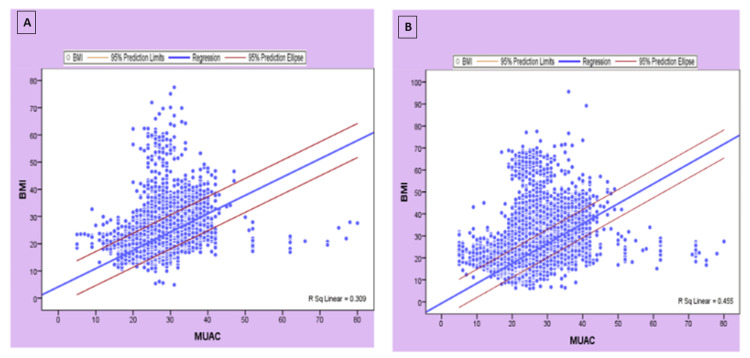
The linear relationship between BMI and MUAC with 95% confidence interval in men (A) and women (B). BMI, body mass index; MUAC, mid-upper arm circumference

The relationship between BMI and MUAC for men (BMI = 4.16 ± 0.67 MUAC) and women (BMI = -0.64 ± 0.90 MUAC) with 95% confidence intervals is presented in Figure 1.

Estimation of MUAC cutoffs against BMI cutoffs

**Figure 2:**
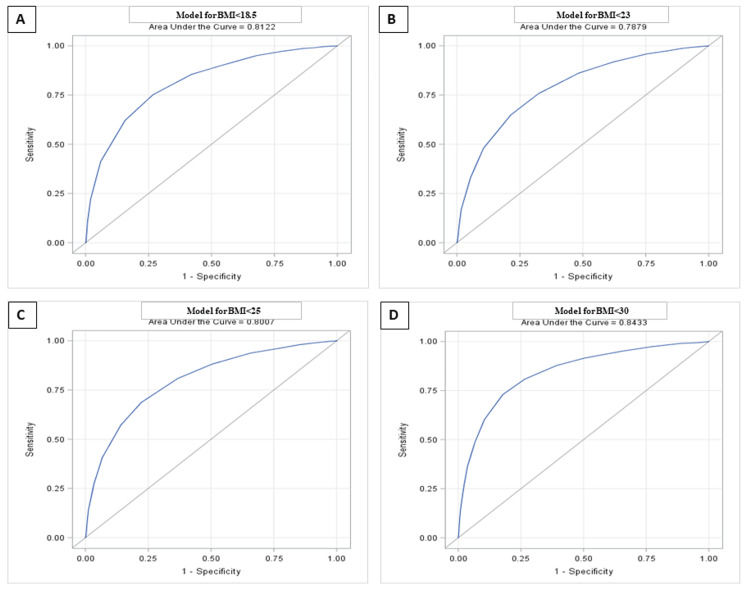
The area under the receiver operating characteristic curve for MUAC based on four different BMI cutoffs: (A) BMI<18.5, (B) BMI<23, (C) BMI<25, and (D) BMI<30. BMI, body mass index; MUAC, mid-upper arm circumference (men).

**Figure 3:**
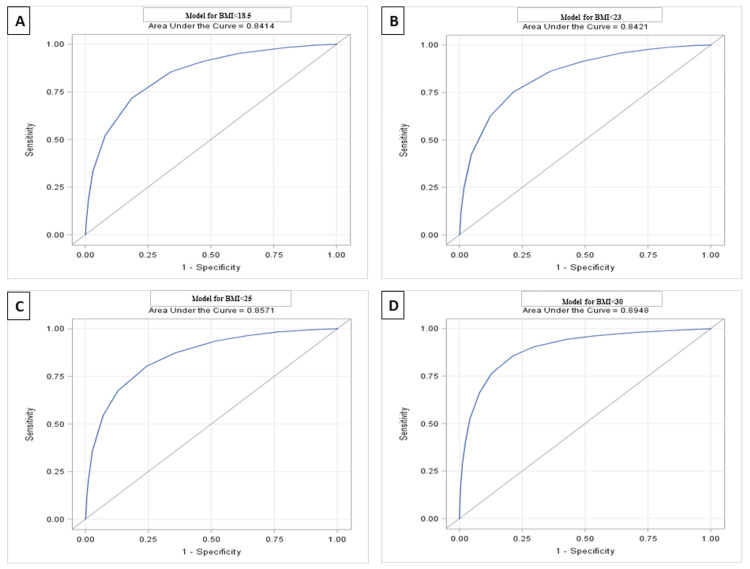
The area under the receiver operating characteristic curve for MUAC based on four different BMI cutoffs: (A) BMI<18.5, (B) BMI<23, (C) BMI<25, (D) and BMI<30. BMI, body mass index; MUAC, mid-upper arm circumference (women).

ROC analysis was carried out to estimate the optimal cutoff for MUAC, and ROC curves were generated. In the present study, the area under the ROC curve (AUROCC) for each of the four BMI values was found to be high: AUROCCs of 0.81, 0.79, 0.80, and 0.84 for BMI 18.5, 23, 25, and 30 kg/m^2^, respectively, for men (Figure 2 and Table 3), and 0.84, 0.84, 0.86, and 0.89 for BMI 18.5, 23, 25, and 30 kg/m^2^, respectively, for women (Figure 3 and Table 3). Coordinate points for each MUAC value were generated.

**Table 3:** AUROCC for mid-upper-arm circumference based on body mass index for men and women. AUROCC, area under the receiver operating curve; CI, confidence interval

Body mass index (kg/m^2^) cutoffs	Men		Women	
	AUROCC (95% CI)	P-value	AUROCC (95% CI)	P-value
18.5	0.81 (0.81-0.82)	0.000	0.84 (0.84-0.84)	0.000
23	0.79 (0.78-0.79)	0.000	0.84 (0.84-0.84)	0.000
25	0.80 (0.79-0.80)	0.000	0.86 (0.85-0.86)	0.000
30	0.84 (0.83-0.85)	0.000	0.89 (0.89-0.90)	0.000

The optimal cutoff values were estimated based on high values of sensitivity and specificity. However, to avoid disagreements, the cutoff values determined by Youden’s index (maxc = {Se (c) + Sp (c) - 1}) were chosen. On the basis of ROC analysis, the MUAC cutoffs for men against the standard BMI cutoffs of 18.5, 23, 25, and 30 kg/m^2^ were approximated to be 25 (Youden's index 0.48), 26 (Youden's index 0.44), 28 (Youden's index 0.46), and 30 (Youden's index 0.55) cm, respectively (Table 4), and the MUAC cutoffs for women were estimated to be 23 (Youden's index 0.53), 25 (Youden's index 0.54), 27 (Youden's index 0.56), and 28 (Youden's index 0.64) cm, respectively (Table 4), as they possess higher values of Youden’s index.

**Table 4:** Evaluation of screening test of standard cutoffs of BMI by MUAC based on the highest Youden’s index. BMI, body mass index; MUAC, mid-upper arm circumference; LR+, likelihood ratio for positive test; LR-, Likelihood RATIO for negative test

BMI (kg/m^2^) cutoffs	Youden's index	MUAC cutoffs (cm)	Sensitivity	Specificity	LR+	LR-
Men						
18.5	0.48	25	0.75	0.73	2.81	0.33
23	0.44	26	0.65	0.79	3.02	0.44
25	0.46	28	0.78	0.69	2.49	0.32
30	0.55	30	0.73	0.82	4.09	0.33
Women						
18.5	0.53	23	0.72	0.81	3.87	0.34
23	0.54	25	0.75	0.79	3.50	0.31
25	0.56	27	0.76	0.80	3.86	0.30
30	0.64	28	0.85	0.78	4.03	0.18

Diagnostic test accuracy of MUAC cutoffs

The diagnostic test accuracy of MUAC cutoffs was assessed based on the sensitivity (SENS), specificity (SPEC), LR+, LR-, rate of false negatives (FN), and rate of false positives (FP) of various cutoffs against the standard BMI cutoffs. The MUAC cutoffs for men and women showed a high range of sensitivity and specificity. Table 4 reports the sensitivity (65-78%) and specificity (69-82%) for men and the sensitivity (72%-85%) and specificity (78%-81%) for women.

Sensitivity is defined as the probability of having a MUAC < cutoff given that BMI is < standard cutoff.

Specificity is defined as the probability of having a MUAC ≥ cutoff given that BMI is ≥ standard cutoff.

Table 4 also depicts the LR+ and LR- of MUAC cutoffs for men and women, respectively. The LR+ for men and women ranged from 2.49-4.09 to 3.50-4.03, respectively, indicating a smaller effect on increasing the probability of malnutrition. The LR- for men and women ranged from 0.32-0.44 to 0.18-0.34, respectively, indicating a moderate effect on decreasing the probability of malnutrition.

LR+ = sensitivity/(1-specificity)

LR- = (1- sensitivity)/specificity

The MUAC cutoffs must take into account the trade-off between failing to capture the entire population in need of services (higher FN rate) and referring too many people who do not require services to the health care system or program (higher FP rate). The combination of FP rate and FN rate is referred to as the “extent of misclassification” and is calculated using MUAC cutoffs instead of standard BMI cutoffs (Table 5).

**Table 5:** Misclassification range of optimal MUAC cutoffs corresponding to BMI cutoffs. The data are represented as n (%). BMI, body mass index; MUAC, mid-upper arm circumference; FP, false positives; FN, false negatives FP = false positives or proportion of individuals without malnutrition who are referred for further screening. FN = false negatives or proportion of individuals with malnutrition that failed to be referred using the MUAC cutoff.

MUAC cutoffs (cm)	BMI (kg/m^2^) cutoffs		Total misclassification (FP+FN) (%)
Men			
	<18.5	≥18.5	
<25	8,273 (8.95)	12,395 (13.40)	18.85
≥25	5,044 (5.45)	66,761 (72.20)	
	<23	≥23	
<26	27,466 (29.70)	3,721 (4.02)	36.18
≥26	29,738 (32.16)	31,548 (34.12)	
	<25	≥25	
<28	50,567 (54.68)	4,202 (4.54)	29.44
≥28	23,023 (24.90)	14,681 (15.88)	
	<30	≥30	
<30	73,656 (79.65)	769 (0.83)	18.1
≥30	15,967 (17.27)	2,081 (2.25)	
Women			
	<18.5	≥18.5	
<23	40,253 (9.24)	28,674 (6.58)	15.01
≥23	36,698 (8.43)	329,829 (75.74)	
	<23	≥23	
<25	167,566 (38.48)	20,671 (4.75)	22.73
≥25	100,071 (22.98)	147,146 (33.79)	
	<25	≥25	
<27	267,044 (61.33)	25,314 (5.81)	20.71
≥27	64,883 (14.90)	78,213 (17.96)	
	<30	≥30	
<28	323,344 (74.25)	3,556 (0.82)	20.87
≥28	87,310 (20.05)	21,244 (4.88)	

## Discussion

The NFHS-4 data revealed a significant increase in obesity/overweight in both adult men and women in the past decade. The burden of undernutrition is prevalent in rural areas, where 20% of men and 23% of women suffer dietary challenges. Anthropometric measurements are not only useful in assessing the nutritional status, growth, and development of individuals but also play a pivotal role in public health programs, as they can be used for screening, goal setting, monitoring, and tailoring interventions. BMI is widely used in clinical practice, research, and public health settings, as it is a simple and quick way to estimate underweight, normal, overweight, or obese with minimal training. Although BMI is a good predictor of health outcomes [20], there is a need to assess other simpler measures, such as MUAC, that could be used in situations such as famine, emergencies, diseased, differently disabled persons, pregnant women, resource-constrained health facilities, and community-level assessments.

We gathered the anthropometric measurements of adult men and nonpregnant women and observed a moderate relationship between BMI and MUAC. Though the public health programs rely on cutoffs rather than the actual value, the current research focused on determining MUAC cutoffs against the standard BMI cutoffs. The ROC-derived MUAC cutoffs discovered reliable agreement with BMI. This validates the findings from previous research conducted in Jharkhand, West Bengal, and other Southeast Asian nations [5,20-25].

Numerous nations and programs have developed the MUAC cutoffs due to the lack of single, widely accepted cutoffs, and paucity of evidence supporting the predictive accuracies of these cutoffs. Therefore, we presented information on the diagnostic test accuracy of specific MUAC cutoffs and showed moderate sensitivity and specificity. These findings support the prediction capability of MUAC for screening nutritional outcomes.

It is well known that the BMI and MUAC are not diagnostic tools and are simply the indicators of nutritional status; the MUAC can be recommended for simplicity. This notion was supported by research on the Sudanese population, which favored the practical application of MUAC over BMI for detecting adult undernutrition in famine [16]. Since the index is the measurement itself in MUAC, it does not require complex mathematical calculations or expensive equipment other than a MUAC four-colored tape (red for severe acute malnutrition, orange for moderate acute malnutrition, yellow for risk of acute malnutrition, and green for well nourished). Furthermore, the MUAC tool can be best equipped in rural areas by frontline health workers as well as in remote areas through field workers with minimal training for community-level screening.

However, there are a few limitations to the current assessment. The NFHS-4 gathered data on arm circumference for the selection of appropriate cuff size for accurate measurement of blood pressure. Although the technique of measurement of arm circumference is similar to MUAC, the data quality remains questionable [26]. Additionally, the study does not take into account the ethnicity and medical condition of the participants. Therefore, further research needs to focus more on the ethnicity and medical condition of the representative sample.

## Conclusions

The present study validates MUAC as a tool that correlates very well with BMI and can be used as a substitute for BMI in resource-constrained health settings and population-level screening. The non-invasive MUAC offers several advantages including accuracy, ease of measurement, and minimal logistical support and training, and can assess the nutritional status even in geographically remote areas. However, few considerations need to be kept in mind as MUAC changes significantly with age. Therefore, different cutoffs must be proposed for different ages. These cutoffs can be of great importance in public health, especially in identifying individuals or populations at risk of malnutrition.
